# Jejunal Diverticulosis Probably Leading to Pylephlebitis of the Superior Mesenteric Vein

**DOI:** 10.1155/2020/2343218

**Published:** 2020-09-16

**Authors:** Julia Bockmeyer, Stephanie Taha-Mehlitz, Nickolaus Heeren, Stefan Ristic, Jürg Metzger, Jörn-Markus Gass

**Affiliations:** ^1^Department of General Surgery, Cantonal Hospital of Lucerne, Spitalstrasse, 6000 Lucerne 16, Switzerland; ^2^Clarunis, Department of Visceral Surgery, University Centre for Gastrointestinal and Liver Diseases, St. Clara Hospital and University Hospital, Basel Kleinriehenstrasse 30 4058, Switzerland; ^3^Department of General Surgery, Cantonal Hospital of Nidwalden, Ennetmooserstrasse, 6370 Stans, Switzerland

## Abstract

Thrombophlebitis of the portal vein (pylephlebitis) is a rare but serious condition with a high mortality rate of 11-50%. A 56-year-old male patient presented with a two-day history of postprandial, colic-like epigastric pain, nausea, fever, chills, and diarrhea. Clinical workup showed peritonism, leukocytosis, and elevated C-reactive protein (CRP). A computed tomography (CT) scan revealed a long-segment, partial thrombosis of the superior mesenteric vein as well as gas in the portal venous system. Additionally, extensive jejunal diverticulosis was present. Pylephlebitis mostly results from intestinal infections, e.g., appendicitis or diverticulitis. We assumed that the patient had suffered from a self-limiting episode of jejunal diverticulitis leading to septic thrombosis. Initially, antibiotic therapy and anticoagulation with heparin were administered. The patient deteriorated, and due to increasing abdominal defense, fever, and hypotension, a diagnostic laparoscopy was performed. Bowel ischemia could be ruled out, and after changing antibiotic therapy, the patient's condition improved. He was discharged without any further complications and without complaints on day 13. An underlying coagulopathy like myeloproliferative neoplasm or antiphospholipid syndrome could be ruled out.

## 1. Introduction

Purulent thrombophlebitis of the superior mesenteric vein and the portal venous system is a rare but serious condition with a mortality rate of 11-50%. The very high mortality rate might be caused by delayed diagnosis due to unspecific symptoms [1–4]. It often occurs in combination with intra-abdominal infection and inflammation like pancreatitis, diverticulitis, or appendicitis. We report the case of a 56-vear-old male patient who suffered from a pylephlebitis most probably originating from jejunal diverticulitis. The thrombophlebitis was treated with antibiotic therapy for 6 weeks and anticoagulation for at least 12 months.

## 2. Case Report

**Figure 1:**
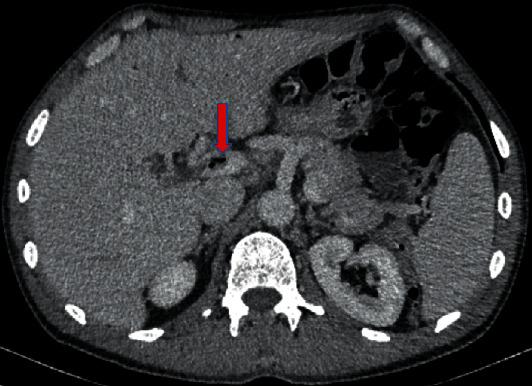
Portal vein thrombosis with intraluminal gas.

**Figure 2:**
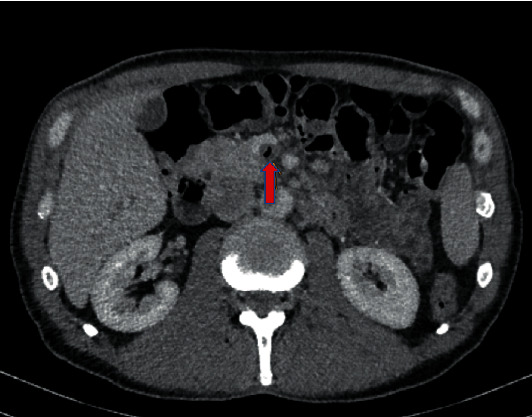
Pneumatosis of the superior mesenteric vein.

A 56-year-old male patient suffered for two days from postprandial, colic-like epigastric pain, nausea, fever, chills, and diarrhea. In the beginning, the symptoms lead to the diagnosis gastroenteritis. His family doctor recommended a CT scan which revealed a long-segment, partial superior mesenteric vein thrombosis as well as gas in the portal venous system (Figures 1 and 2). Additionally, extensive jejunal diverticulosis without signs of diverticulitis was present.

**Figure 3:**
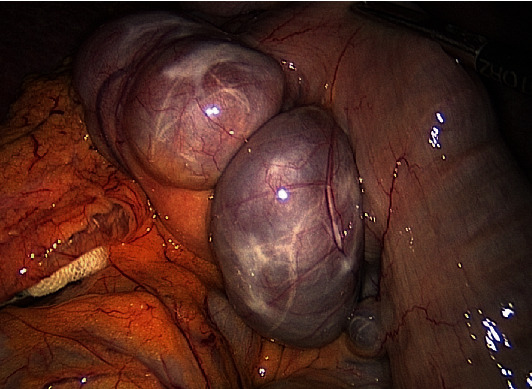
Intraoperative laparoscopic image of the extensive jejunal diverticulosis.

Finally, the patient was transferred to our hospital for further therapy. Clinical workup showed a nonicteric, febrile (38.4°), and normotonic patient with a normofrequent heart rate (RR 109/71; 75 bpm). Clinical examination showed defense and tenderness of the whole abdomen mainly in the epigastric quadrant. Blood samples revealed leukocytosis of 9.2 giga/L and CRP of 119 mg/L. Liver enzymes were in the normal range (ALAT 39 U/L, ASAT 29 U/L). Microbiological analysis of the blood cultures detected polymicrobial bacteremia (*Escherichia coli* and *Granulicatella adiacens*). Antimicrobial therapy with ceftriaxone and metronidazole and anticoagulation with heparin were started. Because the patient's condition deteriorated, antibiotic treatment was changed to piperacillin and tazobactam. Due to increasing abdominal defense, persisting fever, and hypotension, a second CT scan was performed, which could not definitely rule out an underlying bowel ischemia. A diagnostic laparoscopy showed no signs for a bowel ischemia, ongoing diverticulitis, or any other intra-abdominal infection (Figure 3). Postoperatively, the patient stayed in the intensive care unit for one day due to hypotension. During the further course, recurrent fever episodes occurred. Repetitive blood samples showed polymicrobial bacteremia with *Enterobacter cloacae* despite ongoing antibiotic therapy. Antimicrobial therapy was adapted to these results (cefepime and metronidazole), and the patient stayed afebrile. At the time of discharge, the antibiotic therapy was changed to moxifloxacin 400 mg per day by mouth for further four weeks. During hospital stay, several hematological tests were performed, on the one hand, to rule out any hematologic etiology such as hypercoagulopathy (e.g., myeloproliferative neoplasm or antiphospholipid syndrome) and, on the other hand, to start anticoagulation. Molecular-biological and genetic blood tests ruled out JAK2 V61F, MPL W515L/K, and calreticulin mutations, thus no hint for a myeloproliferative neoplasm.

Elevated anti-cardiolipin IgM antibodies (26 MPL-U/mL [<10]) were suspicious for antiphospholipid syndrome. Diagnosis could not be confirmed since blood results three months later showed standard values (anti-cardiolipin IgG antibodies: 1.6 GPL-U/mL, anti-beta2-glycoprotein 1 IgG: 0.8 U/mL, anti-beta2-glycoprotein 1 IgM: 0.3 U/mL, and lupus anticoagulants negative). On discharge, anticoagulation with Marcoumar was started and recommended for at least 12 months.

## 3. Discussion

Pylephlebitis is a rare but serious condition with significant morbidity and mortality of 11-50% [1–4]. Pathophysiologically, pylephlebitis often starts with bacteremia and thrombophlebitis in small veins, which drain the region of the intra-abdominal infection. This can secondarily lead to an infection of the larger mesenteric veins and the whole portal venous system [5]. Furthermore, thrombophlebitis in small veins can lead stepwise to hypercoagulopathy and finally to septic embolism [4, 6, 7]. The superior mesenteric vein is involved in 42% followed by the portal vein (39%) and splenic vein (12%) [4, 8]. Thrombosis of mesenteric veins is often accompanied with bowel ischemia and infarction and thus can finally lead to death [2, 3, 5]. The underlying abdominal infection is mostly diverticulitis or appendicitis [1, 4, 9]. Diagnosis of pylephlebitis is challenging since clinical presentation and findings in clinical workup are often nonspecific (e.g., leukocytosis or elevated liver enzymes) and thrombosis may occur with a delay of up to 30 days after abdominal infection [2, 3, 8, 10–12]. Therefore, a correct diagnosis might be delayed or even missed [10]. Our patient presented with an elevated CRP of 119 mg/L, slight leukocytosis (9.2 giga/L), and normal liver enzymes. Clinical examination was initially typical for gastroenteritis. The further clinical course was untypical; therefore, a CT scan was performed. If diagnosis after abdominal CT scan still remains unclear, an MRI is recommended [4, 13]. In previously reported cases, blood samples were positive in 23-88% [1, 3–5, 8]. Frequently, isolated microorganisms are *Bacteroides fragilis*, *Escherichia coli*, *Proteus mirabilis*, *Clostridium* spp., *Klebsiella* ssp., *Streptococcus pneumonia*, and *Aerobacter* spp. [4, 14] In our patient *Escherichia coli*, *Granulicatella adiacens*, and *Enterobacter cloacae* bacteraemia were detected. Once pylephlebitis has been diagnosed, antibiotic therapy and anticoagulation should be started immediately. In case of suspected bowel ischemia, a diagnostic laparoscopy should be performed [1, 2, 4, 15]. While antibiotic therapy is strongly recommended, anticoagulation is still under debate [1, 15, 16]. Some authors recommend anticoagulation only if there are any risk factors such as clotting factor deficiencies or neoplasms. Duffy et al. recommended anticoagulation therapy depending on the exact localization of the thrombosis and the presence of bowel ischemia as well as in patients who did not respond to antibiotic and/or surgical therapy [3, 15, 17]. On the other hand, according to Plemmons et al. and Kanellopoulou et al., patients who were treated with both antibiotics and anticoagulation had better outcomes compared with those who were treated with antibiotics alone [1, 8]. For anticoagulation, LMWH and warfarin were most frequently used [1, 4]. Recommendations for the duration of antibiotic therapy vary. Nevertheless, it should be continued for a minimum of 4 weeks in most cases to avoid complications such as hepatic abscesses [1, 15, 18]. In our case, antibiotic therapy was administered for 6 weeks and anticoagulation for 12 months.

## 4. Conclusion

Pylephlebitis is a rare but serious condition. Mostly, intra-abdominal infections like diverticulitis or appendicitis are the underlying reasons. Because of the high mortality rate, it should always be kept in mind when intra-abdominal infection is present and the patient deteriorates despite correct therapy. Individual treatment with an interdisciplinary approach addressing the extent of the thrombosis, microbiological results, and personal risk factors are the cornerstones of therapy.
